# High-density lipoprotein contribute to G0-G1/S transition in Swiss NIH/3T3 fibroblasts

**DOI:** 10.1038/srep17812

**Published:** 2015-12-07

**Authors:** Fabrizio Angius, Stefano Spolitu, Sabrina Uda, Stefania Deligia, Alessandra Frau, Sebastiano Banni, Maria Collu, Simonetta Accossu, Clelia Madeddu, Roberto Serpe, Barbara Batetta

**Affiliations:** 1 Unit of Experimental Medicine, University of Cagliari, Cagliari, Italy; 2 Divisions of Physiology, University of Cagliari, Cagliari, Italy; 3 Neuroscience and Clinical Pharmacology, University of Cagliari, Cagliari, Italy; 4 Department of Biomedical Sciences, Department of Medical Sciences “Mario Aresu”, University of Cagliari, Cagliari, Italy

## Abstract

High density lipoproteins (HDLs) play a crucial role in removing excess cholesterol from peripheral tissues. Although their concentration is lower during conditions of high cell growth rate (cancer and infections), their involvement during cell proliferation is not known. To this aim, we investigated the replicative cycles in synchronised Swiss 3T3 fibroblasts in different experimental conditions: i) contact-inhibited fibroblasts re-entering cell cycle after dilution; ii) scratch-wound assay; iii) serum-deprived cells induced to re-enter G1 by FCS, HDL or PDGF. Analyses were performed during each cell cycle up to quiescence. Cholesterol synthesis increased remarkably during the replicative cycles, decreasing only after cells reached confluence. In contrast, cholesteryl ester (CE) synthesis and content were high at 24 h after dilution and then decreased steeply in the successive cycles. Flow cytometry analysis of DiO-HDL, as well as radiolabeled HDL pulse, demonstrated a significant uptake of CE-HDL in 24 h. DiI-HDL uptake, lipid droplets (LDs) and SR-BI immunostaining and expression followed the same trend. Addition of HDL or PDGF partially restore the proliferation rate and significantly increase SR-BI and pAKT expression in serum-deprived cells. In conclusion, cell transition from G0 to G1/S requires CE-HDL uptake, leading to CE-HDL/SR-BI pathway activation and CEs increase into LDs.

A high rate of cholesterol synthesis, uptake and esterification in proliferating tissues, accompanied by reduction of plasma cholesterol-HDL, has been extensively described during tumour growth^1^,^2^,^3^,^4^,^5^,^6^. The higher requirement of cholesterol has been attributed to its structural and functional properties in cell membrane biogenesis, while the elevated content of CEs was mainly ascribed to the fact that membrane cholesterol, rather than being delivered to HDL, is preferentially shifted to ER for esterification by ACAT. Therefore, cellular accumulation of CE may be considered an additional store to provide cholesterol during sustained membrane biogenesis. We have reported that CEM-CCRF, a lymphoblastic cell line characterised by high rate of CE synthesis^7^, further increase their content by obtaining preformed CEs from HDL^8^. Similarly, in a macrophage tumour-like cell line P388D1, the content of CEs and CE-HDL uptake, probably mediated by SR-BI protein, was further induced by microbial stimulus^9^. SR-BI is a membrane protein involved in cell cholesterol efflux in peripheral tissues but responsible for CE-HDL uptake in specialised organs with high requirement of cholesterol, like adrenal glands and liver. However, SR-BI expression has been reported to be elevated in several tumour cell types^10^,^11^. For this reason, we Hypothesised that the main function of HDL, related to reverse cholesterol transport activity, may change when peripheral requirement of cholesterol increases (i.e., infections and tumours) being their plasma levels regulated by cell request^9^. Beside tumours, increase of macrophage-CE has been described in atherosclerotic lesions, but the possibility that these neutral lipids are also controlled during proliferation of normal cells has been completely neglected. Indeed, the scarcity of studies investigating a possible role of CEs in the pathway involved in cell proliferation may be due to the bulk of literature associating their accumulation with pathological conditions (i.e., cancers and atherosclerosis). We have previously observed an increase of CE synthesis during the growth of aortic primary smooth muscle cells^12^. Moreover CE content in LDs is elevated in exponentially growing mouse 3T3 fibroblasts, while being virtually absent in confluent quiescent cells^13^. Recently, the HDL receptor SR-BI has been demonstrated to activate phosphorylation of the AKT-dependent mitogen signal, suggesting a correlation between CE-HDL uptake and the proliferative signaling cascade^14^,^15^,^16^. It is then reasonable to Hypothesise that changes in CEs pathway is present also during normal cell proliferation. For this reason, we investigated CE metabolism during the growth of synchronised mouse 3T3 fibroblasts.

## Results

### Cell cycle distribution and proliferation after contact-inhibition loss

**Figure 1:**
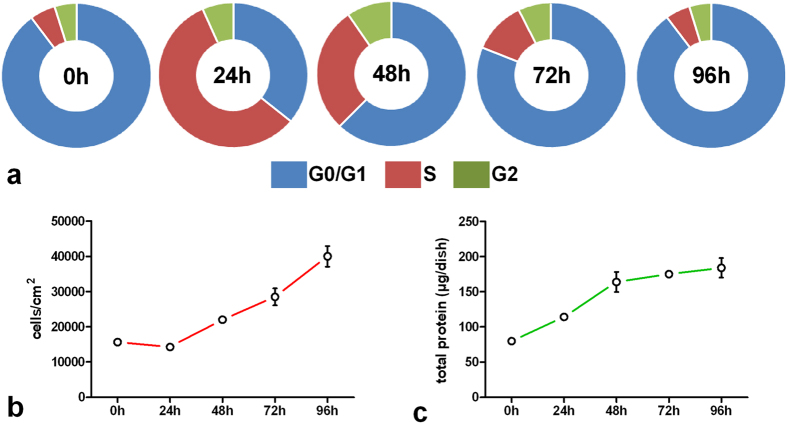
Cell cycle distribution after contact-inhibition loss. Contact-inhibited 3T3 Swiss mouse fibroblasts were diluted at 1.5 × 10^4^cells/cm^2^ (exponential growth) up to the concentration of about 4.0 × 10^4^cells/cm^2^ (stationary growth), and harvested at the indicated times. (**a**) For FACS analysis, experiments were performed as reported in Materials and Methods. Each sample was in triplicate and experiments were performed at least twice. Cell cycle distribution is reported as percentage of cells in each discrete phase. At each time an aliquot of cells was used for cell count (**b**) and another aliquot for protein determination (**c**). Data are given as mean ± SE. *p < 0.05 vs all (t-student test, unpaired two-tailed).

Figure 1 (panel a) represents the phase distribution of cells at 0, 24, 48, 72 and 96 h after loss of contact-inhibition. At quiescence (0 h), most of cells were in G0/G1 (90%), while 5% were at S and G2. At 24 h, cell percentage, increased at S and G2 (57 and 7%, respectively), while 36% of cells were in G0/G1. At 48 h, 62% of cells were in G0/G1, while 28 and 10% were in S and G2, respectively. At 72 h, most of cells were at G0/G1 (81%), whereas the percentages found at S (12%) and G2 (7%) were lower compared to 24 h proliferating cells. At 96 h, cell cycle distribution was similar to that found at 0 h. Cell number doubled at 48, 72 and 96 h (Fig. 1b), whereas the higher content of total protein at 24 h, compared to 0 h, suggests that cells were mainly in G1 rather than G0 phase (Fig. 1c).

### CE, TG and cholesterol synthesis after contact-inhibition loss

**Figure 2:**
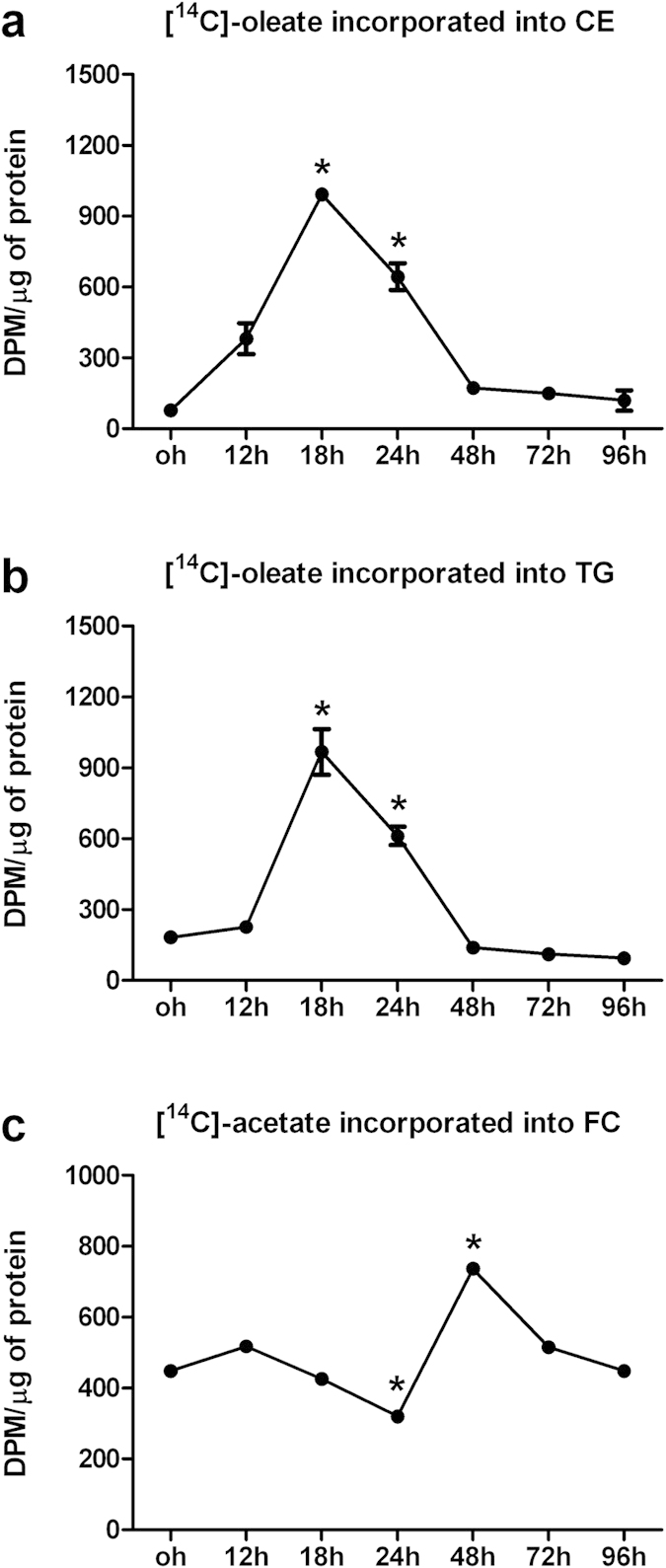
Cholesteryl ester, trygliceride and cholesterol synthesis after contact-inhibition loss. Cholesteryl ester (**a**), triglyceride (**b**) and cholesterol (**c**) synthesis were evaluated at the indicated times after diluting cells at exponential growth and followed up to quiescence. Cells were diluted at 1.5 × 10^4^cells/cm^2^ (exponential growth) up to the concentration of about 4.0 × 10^4^cells/cm^2^ (stationary growth). Cells were incubated for 4 h in medium containing [^14^C]-oleate bound to bovine serum albumin (BSA) (CE and TG synthesis) or 3 h with 37 KBq/ml of [^14^C]-sodium acetate (FC synthesis). After incubation, cells were washed with ice-cold PBS, and lipids were extracted with cold acetone and separated by TLC and incorporation of [^14^C]-oleate and sodium [^14^C]-acetate were measured as described in Methods. An aliquot of cell lysate was processed for protein content. All data are expressed as the rate of [^14^C]-oleate or sodium [^14^C]-acetate incorporation into CEs, TGs and FC for μg of protein, respectively. Each experiment was carried out at least twice in triplicate and the results are given as mean ± SE. *p < 0.05 vs all (t-student test, unpaired two-tailed).

Figure 2 shows the CE, TG and cholesterol synthesis evaluated through all cell cycles from contact inhibition loss up to quiescence. The rate of cholesterol esterification (Fig. 2a) was found significantly higher at 18 and 24 h (p < 0.05) from contact loss, whereas it was almost absent at 48, 72 and 96 h after dilution. TG synthesis (Fig. 2b) demonstrated the same trend as CE synthesis, showing higher values at 18 and 24 h (p < 0.05). Although to different extent, cholesterol synthesis (Fig. 2c) was variable during all replicative cell cycles, showing the lowest value at 24 h and peaking at 48 h after contact inhibition loss (p < 0.05).

### Flow cytometry analysis of DiO-HDL and radiolabeled [^14^C]-CE-HDL uptake in cycling and quiescent fibroblasts

**Figure 3:**
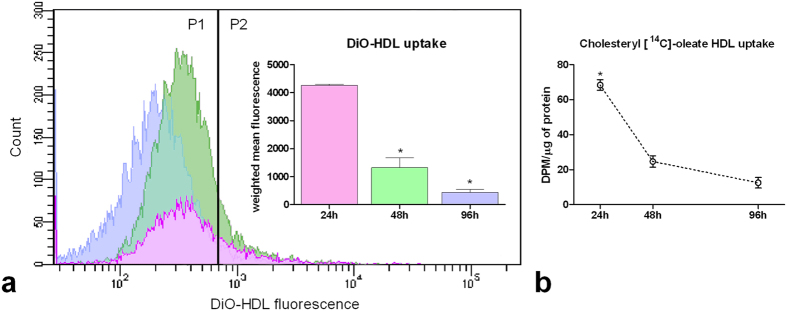
FACS analysis of DiO-HDL fluorescence and radiolabeled cholesteryl [^14^C]-oleate HDL uptake in 24, 48 and 96 h cultured 3T3 Swiss mouse fibroblasts. Panel a: shows the FACS diagram of fluorescence signal related to DiO-HDL uptake in 24, 48 and 96 h fibroblasts after contact loss. Cells were incubated with DiO-HDL and prepared for FACS analysis as reported in Material and Methods section. Each experiment was carried out at least twice and the relative graph shows the weighted mean value ± SE of fluorescence evaluated in positive cells (P2) normalized to the entire population (P1 + P2). At least 10000 cells for each group were analysed. *p < 0.01 vs 24 h (t-student test, unpaired two-tailed). Panel b: cells were incubated with 100 mM [^14^C]-CE-HDL in LPDS 24 h before the harvesting times. Cells were harvested and lipids extracted as described in Material and Methods section. Each experiment was carried out at least twice in triplicate and the results are given as mean ± SE. *p < 0.05 vs all (t-student test, unpaired two-tailed).

Figure 3a shows the curve distribution of DiO-HDL fluorescence at 24, 48 and 96 h from cells dilution. As reported in the relative graph the fluorescent signal is significantly higher at 24 h compared to the other time points (p < 0.01). Accordingly, as shown in Fig. 3b, radiolabeled CE-HDL uptake was remarkably higher at 24 h after the contact loss, steeply decreasing at 48 h and reaching the lowest value at the resting state after 96 h (p < 0.05).

### DiI-HDL uptake, DiI-LDL binding, SR-BI detection and lipid distribution in cycling and quiescent fibroblasts

**Figure 4:**
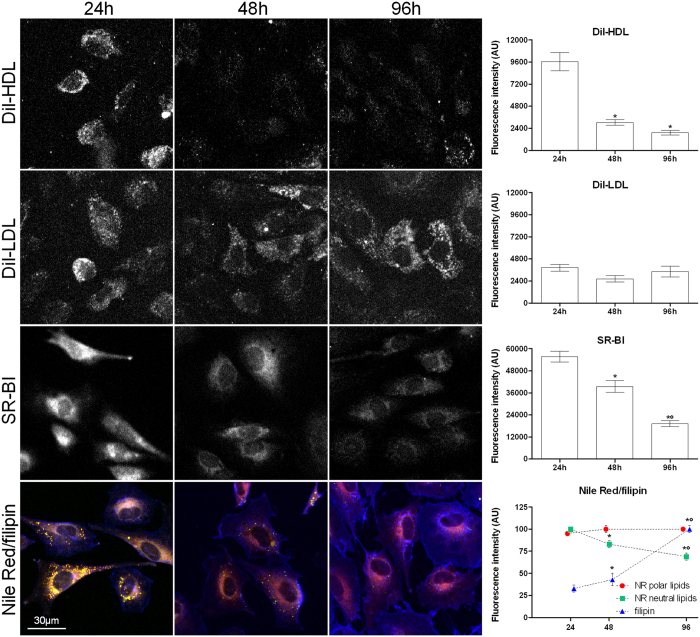
DiI-HDL uptake, DiI-LDL binding, SR-BI immunodetection and Nile Red/filipin double stain in 24, 48 and 96 h cultured 3T3 Swiss mouse fibroblasts. The figure shows DiI-HDL uptake, DiI-LDL binding, SR-BI immunocytofluorescence and merged color images obtained by Nile Red/filipin double staining, that show polar lipids and cytoplasmic membranes (NR polar lipids) in red, LDs (NR neutral lipids) in green/yellow and plasma membrane cholesterol (filipin) in blue. All detections were tested on fibroblasts cultured for 24, 48 and 96 h after contact loss in standard medium. Each experiment was carried out at least twice and the relative graphs and histograms show the mean values ± SE for each single fluorescence emission signal evaluated in at least 200 cells for each group. Bar is 30 μm. *p < 0.05 vs 24 h; °p < 0.05 vs 48 h (ANOVA, Fischer’s LSD as post hoc test).

As shown in Fig. 4, CE-HDL uptake was very active at 24 h, while being almost absent 48 and 96 h after dilution, as confirmed by image analysis (p < 0.05). The CE-HDL uptake greediness by proliferating cells was highly evident when compared to quiescent cells (see movies). Conversely, LDL binding (Fig. 4) did not show significant differences when compared to the other groups. SR-BI, revealed by immunofluorescence, was remarkably higher at 24 h (p < 0.05), progressively decreasing at 48 and 96 h, following the same trend as for DiI-HDL. Although NR polar lipid (red circles) fluorescence did not change over the groups, the image analysis of NR neutral lipids (green squares) showed a higher signal at 24 h (p < 0.05) that decreased steeply at the later points. The figure shows also that the plasma membrane cholesterol content, demonstrated by filipin staining, was significantly increased at 96 h (blue triangles) (p < 0.05).

### FC and CE content in 3T3 fibroblasts

HPLC analysis (table 1) revealed that FC content did not show significant changes between the two experimental groups. Conversely, the intracellular CE content was more than 2-folds higher in 24 h exponentially growing cells compared to quiescent cells (p < 0.05). The remarkable increase of CE content affected the total cholesterol (TC) content as well as the CE/TC ratio that was significantly higher in diluted cells compared to confluent cells (p < 0.05).

### Scratch wound assay

**Figure 5:**
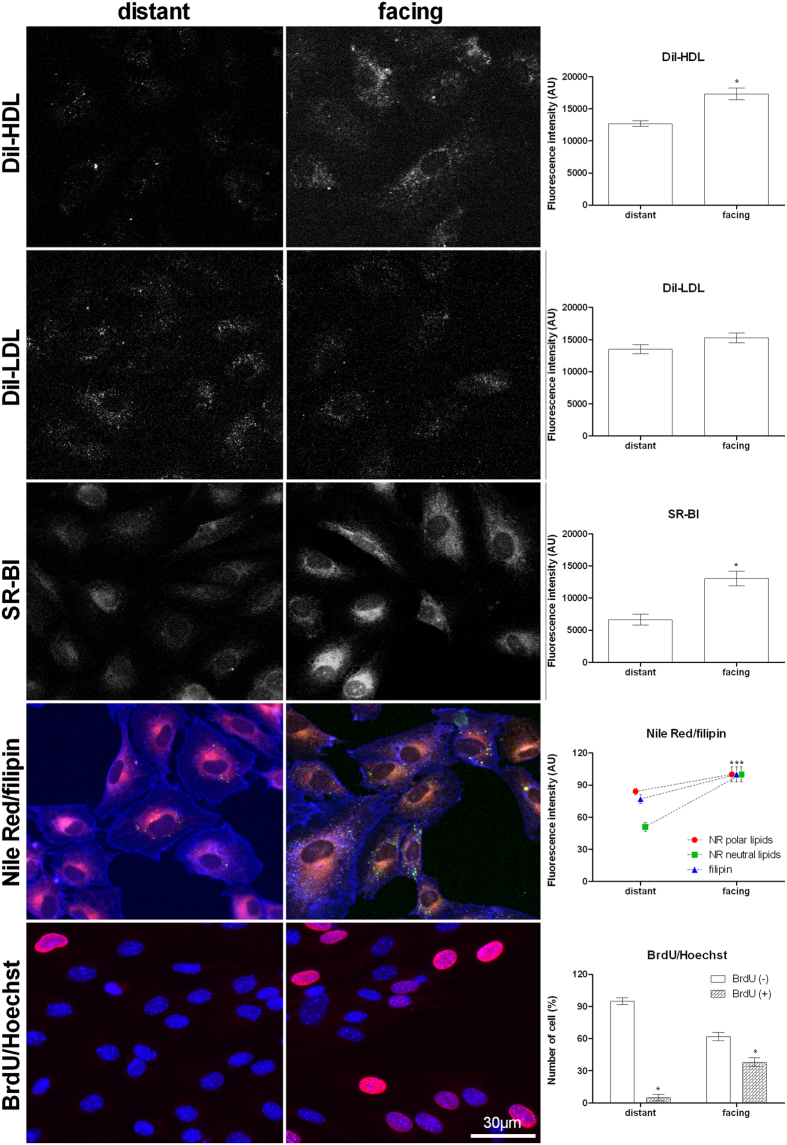
Scratch wound assay. The figure reports DiI-HDL uptake, DiI-LDL binding, SR-BI immunocytofluorescence detection, the merged colour images obtained by Nile Red/filipin double staining, that show polar lipids and cytoplasmic membranes in red, LDs in green/yellow and plasma membrane cholesterol (filipin) in blue. The figure shows also BrdU (red) incorporated in DNA (Hoechst, blue). All detections were performed in distant (quiescent) and facing (proliferating) 3T3 Swiss mouse fibroblasts after 24 h from scratch-wound. Each experiment was carried out at least twice and the relative graphs and histograms show the mean values ± SE for each single fluorescence emission signal evaluated in at least 200 cells for each group. Bar is 30 μm. *p < 0.05 vs distant (ANOVA, Fischer’s LSD as post hoc test).

The Fig. 5 shows DiI-HDL uptake, DiI-LDL binding, SR-BI immunostaining and neutral lipid content (NR) in proliferating (facing) and quiescent (distant) cells in the same culture dish after 24 h from the scratch wound. DiI-HDL uptake was present in cells facing the wound, whereas it was largely absent in contact-inhibited cells (distant). DiI-LDL binding was similar in contact-inhibited cells and in those facing the wound. SR-BI immunostaining followed the same trend as DiI-HDL, being higher in cells in the wound area. Neutral lipids (NR) and resumption of proliferation (BrdU/Hoechst) were present in cells facing the wound but were almost absent in distant quiescent contact-inhibited cells.

### Cell cycle distribution, SR-BI, pAKT and pERK protein expression in serum-deprived cells entering the cycle after 24 h treatment with FCS, HDL or PDGF

**Figure 6:**
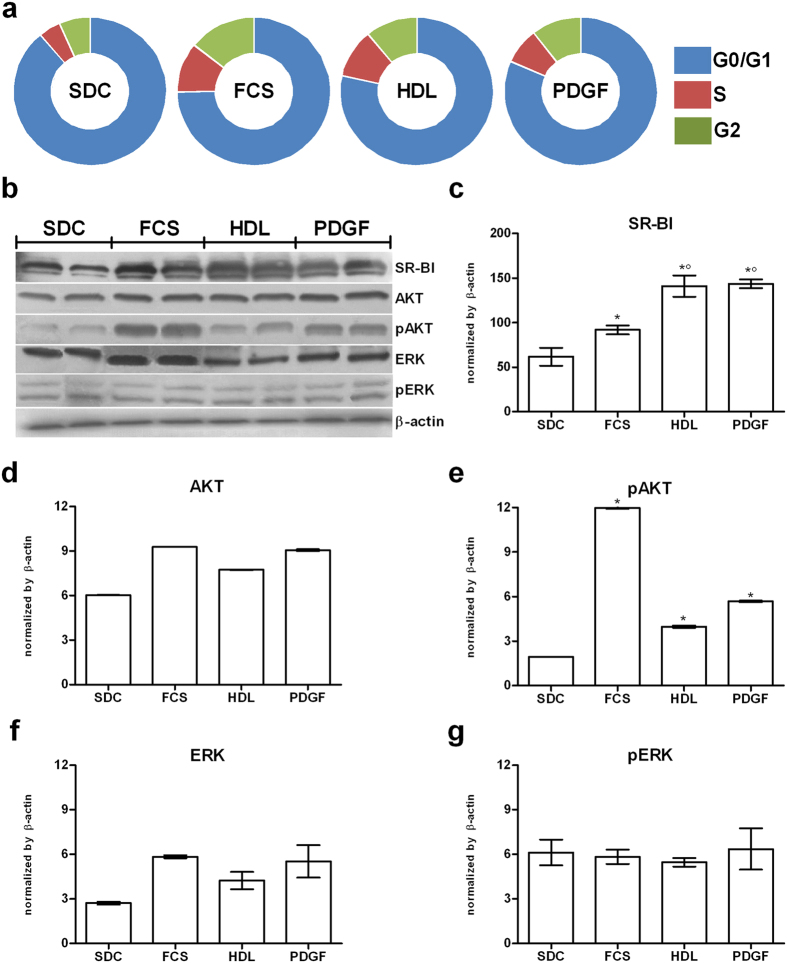
Cell cycle distribution, SR-BI, AKT and ERK protein expression in serum-deprived cells entering the cell cycle after 24 h treatment with FCS, HDL or PDGF. Sub-confluent cells were cultured for 24 h in serum-deprived medium (SDC). Then treated for further 24 h with FCS, HDL or PDGF, respectively. At the end of experiments cells were harvested and processed as required for cell cycle analysis by FACS (**a**) and western blotting (**b**), respectively. For details see Materials and Methods. The panel (**c**–**g**) show the densitometric analysis of SR-BI, AKT, pAKT, ERK and pERK bands, respectively. Data were reported as mean ± SE of three different experiments. *p < 0.05 vs SDC; °p < 0.05 vs FCS; ^p < 0.05 vs HDL (ANOVA, Fischer’s LSD as post hoc test).

Figure 6a depicts the cell cycle distribution in 24 h serum-deprived cells treated for 24 h with FCS, HDL or PDGF. As shown in the figure, most of serum-deprived cells (SDC) were in G0/G1 (89%), whereas 5 and 6% were in S and G2, respectively. At 24 h from treatment with FCS an increase of S and G2 was seen (11 and 14%, respectively). Similarly, the percentage of HDL treated cells was increased in S and G2 (both 11%). After PDGF treatment, 8% of cells were in S, whereas 11% were in G2. Accordingly, densitometric analysis of pAKT protein expression bands (Fig. 6e) detected a significant increase in FCS and HDL treated cells (p < 0.05). SR-BI protein expression (Fig. 6c), even if to different extent, was increased following each treatment (p < 0.05). No differences were found in pERK protein expression at 24 h (Fig. 6g).

## Discussion

High CE content, mainly associated with pathological conditions (cancer, infections and atherosclerosis), has been related to a high rate of cholesterol esterification and to the reduction of HDL capacity in removing peripheral cholesterol^17^,^18^,^19^. In tumour tissues, this phenomenon has been attributed to the need of a continuous supply of cholesterol for membrane biogenesis. Recent studies have demonstrated that CEs are directly taken up from HDL in tumour and inflammatory cells, suggesting that HDL, rather than removing, may supply CEs to the cells when more CEs are required^8^,^9^. Thus, the possibility of a more general involvement of CEs in cell growth, in particular CE-HDL, has been scarcely considered, with CEs metabolism in normal proliferating cells almost completely ignored. In this study, we report that the passage of quiescent normal fibroblasts to the replicative cycle presents peculiar modifications of CE metabolism, where HDL plays a major role in regulating CE content. These modifications seem to be mainly restricted to the passage of cells from G0 to G1/S phases, whereas they are almost absent in the following cycles up to the subsequent G0 phase induced by contact-inhibition. Indeed, in the present study, flow cytometry analysis of DiO-HDL, definitely shows a significant increase of fluorescent signal in 24 h growing compared to the following cycling and resting cells. We obtained similar results when cells were incubated with radiolabeled CE-HDL. Accordingly, a remarkable increase in CE synthesis and CE-HDL uptake is found 24 h after triggering the passage from G0 to G1/S. These effects were accompanied by a remarkable increase in LDs, even if it is likely that the simultaneous high rate of TG synthesis contribute to increase the LD content. Similarly, the amount of the SR-BI membrane protein was found increased at the same time. Although this protein generally removes excess cholesterol from peripheral cells and delivers it to HDL, its role is devoted to catching preformed CEs in specialised organs (i.e., adrenal glands and liver), where storage of cholesterol is needed for hormone or bile acid synthesis^20^,^21^. However, high expression of this protein has been also described in tumour cells and is thought to be a response to the need of FC for membrane biogenesis^10^,^11^,^22^,^23^. In this study, we demonstrated that fibroblasts stimulated to proliferate show an increase of CEs, stored in LDs, obtained from HDL via SR-BI but, differently from cancer cells, this phenomenon seems to be restricted to the first cell cycle, and disappeared subsequently in G0-G1/S-induced quiescent cells. On the contrary, as reported, cholesterol synthesis and LDL binding are present in all replicative cycles up to quiescence^24^,^25^. The different route undertaken by HDL probably driven by an increased peripheral CE requirements, has been reported in tumours and infections and supported by the fact that reduction of CE-HDL precedes the modifications of the apoprotein component (i.e. apo A1)^26^. Differently from *in vivo*, the *in vitro* studies did not consent to detect nanomolar changes in lipid HDL content but, as shown in the movies attached to this article, the CE-HDL uptake is almost absent in quiescent cells, whereas the fluorescent dye is actively and quickly internalized in exponentially growing cells. Thus, the count of cell number and FACS analysis of cell cycle distribution evaluated at all time points, showed that cells, even if to different extent, were still cycling up to 72 h. Similar results were obtained in both ways that we used to sychronise cells in G0 phase: (i) contact-inhibition and (ii) the scratch wound method, in which both quiescent (distant) and growing cells (facing) can be observed simultaneously in the same culture dish. Also in the latter condition, the cells facing the scratch after 24 h were synthesizing DNA, as demonstrated by BrdU nuclei staining, and showed a higher content of LDs and CE-HDL uptake, and increased SR-BI immunodetection when compared to those distant from the scratch. SR-BI activation has been also involved in signal transduction common with proliferation and often associated with increase of the pAKT and pERK signal pathway^27^. In the experiments with synchronised cells for 24 h in serum-deprived medium and treated for a further 24 h in the presence of FCS, HDL or PDGF, FACS analysis showed that HDL exerted the same mitogenic activity as PDGF although this effect was lower than that exerted by FCS. In our experimental conditions, either HDL and PDGF-treated cells, showed a remarkable increase of SR-BI protein expression, suggesting that the activation can also be dependent upon the mitogenic stimulus. A mitogenic role of HDL has been reported in different cell lines^15^,^16^,^22^,^27^. This activity has been considered to be mediated by the binding of the lipoprotein with its SR-BI membrane receptor^10^,^11^, as the binding activates the same proliferative pathway induced by mitogens (i.e. PDGF) such as inducing phosphorylation of signal transduction pathways^16^. However, serum-deprivation followed by addition of different mitogens is not the best way to perform this kind of study, because of the small number of cycling-induced cells and probably the different effect on the transduction pathway exerted by mitogens. In fact, our hypothesis seems to be supported by the fact that SR-BI and pAKT expressions are apparently dependent upon the mitogens used. No difference was found in pERK protein expression in our experimental condition but, it is worth noting that these signaling cascade factors should be evaluated shortly, after the mitogen signal. Thus, we have concluded that the synchronisation obtained by contact inhibition seems more efficient and, at the same time, more similar to physiological condition. We can hypothesise that the CE-HDL/SR-BI/LDs pathway may be tightly involved in the mitogenic response mainly present during the G0 to G1/S transition, being almost absent in the subsequent cell cycles. On the contrary, studies conducted in tumour cells demonstrated that the increase of SR-BI protein expression is evident during all replicative cycles. In previous studies, we have observed an increased rate of cholesterol esterification and CE accumulation in proliferating liver induced with lead nitrate or in response to partial hepatectomy^28^. A notable increase of CEs accompanied by a consistent reduction of HDL was evident mainly in the first round cycle (24–48 h). In contrast, cholesterol and DNA synthesis were present up to the recovery of the liver size (about 1 week). Despite the intense research on HDL endocytosis and the selective lipid transfer^29^,^30^,^31^,^32^,^33^, to date SR-BI is still considered the most responsible for the selective CE uptake from HDL, whereas CE intracellular movements from plasma-membrane to the LDs are completely unknown. Moreover, as other proteins (i.e., hepatic lipase, lipoprotein lipase) have been proposed to mediate HDL selective CE uptake, we cannot exclude their involvement also during the cell cycle^34^,^35^,^36^,^37^. To conclude, our data strongly suggest that in normal cells, the pathway leading to CE increase is related to the passage from quiescence to proliferation and is achieved mainly through CE-HDL/SR-BI/LDs pathway, which remains continuously active in cancer cells. It remains to be established whether CE storage simply supplies cholesterol requirements in the following cycles or if it is, in some way, related to the control of cell proliferation itself. Identification of the differences in delivering CEs in physiological and pathological proliferation by HDL would open up their use as a promising tool for delivering lipophilic drugs preferentially targeting cancer cells, as already suggested^38^,^39^.

## Materials and Methods

### Cell cultures

Stock culture of Swiss 3T3 mouse fibroblasts (ATTC, Rockville, MD) were cultured in DMEM medium supplemented with 10% fetal bovine serum (FBS), 100 U/ml penicillin, 100 μg/ml streptomycin, 25 mM HEPES and 2 mM L-glutamine at 37 °C in an incubator with a humidified atmosphere of 95% air and 5% CO_2_. Twice weekly, confluent cultures (about 4.0 × 10^4^cells/cm^2^) were trypsinized and seeded at the growing concentration of 1.5 × 10^4^cells/cm^2^. For analyses, fibroblasts were synchronised at quiescence and G0 cells were induced to G1/S entry by two ways. (i) Confluent contacted-inhibited fibroblasts, were trypsinized and re-cultured at the growing concentration of 1.5 × 10^4^cells/cm^2^, cells were harvested at 0, 24, 48 and 96 h after dilution. To visualise, in the same culture, possible differences determined by proliferative stimulus exerted by contact loss, we have utilised the scratch wound assay. Specifically, the confluent cell layer was scraped along a middle line using a pipette tip as previously described^13^, and cells in the scratching line were observed after 24 h. (ii) In sub-confluent cultures medium was removed, and cells were made quiescent by culturing them in presence of serum-free medium for further 24 h (SDC). Afterwards, fibroblast entry into cell cycle was induced with FCS (10%), HDL (100 μg/ml; Bioquote Ltd., York, UK) or PDGF-BB (25 ng/ml; Sigma-Aldrich, Milan, Italy) and harvested after an additional 24 h.

### Determination of cholesterol esterification and triglyceride synthesis from [^14^C]-oleate

For measuring CE and triglyceride (TG) synthesis, cells were grown in 25 cm^2^ flasks and harvested at the indicated times. Cells were incubated for 4 h in medium containing [^14^C]-oleate bound to bovine serum albumin (BSA). To prepare the oleate-BSA complex, 3.7 MBq of [^14^C]-oleic acid in ethanol (specific activity 2.035 GBq/mmol) was mixed with 1.4 mg KOH and the ethanol evaporated. PBS (1.5 ml) without Ca^2+^ and Mg^2+^, containing 4.24 mg BSA (fatty acid-free) was added and the mixture shaken vigorously. This solution was added to each well for a final concentration of 74 KBq/ml. After incubation, trypsinized cells were washed with ice-cold PBS and lipids extracted with acetone. Neutral lipids were separated by TLC as below, and incorporation of [^14^C]-oleate into CEs and TGs was measured^40^. An aliquot of cell lysate was processed for protein content^41^.

### Determination of cholesterol synthesis from [^14^C]-acetate

Cholesterol synthesis was evaluated at the indicated times after diluting cells at 1.5 × 10^4^cells/cm^2^ (exponential growth) up to the concentration of about 4.0 × 10^4^cells/cm^2^ (quiescence). To determine the rate of cholesterol synthesis, 3 h before the harvesting times cells were incubated with 37 KBq/ml of sodium [^14^C]-acetate. After incubation cells were separated by centrifugation and collected. Lipids were extracted with cold acetone and neutral lipids separated by thin layer chromatography (TLC) Kiesegel plates using a solvent system containing heptane/isopropylether/formic acid (60:40:2 v/v/v). As for CEs and TGs, free cholesterol (FC) bands were identified by means of comparison with standards running simultaneously with samples, and visualised using iodine vapor^7^. For counting, bands were excised and added directly to counting vials containing Ultima Gold. All incubations were carried out in triplicate and the results of individual experiments are given as mean values variation between triplicates was less than 10%. All data are expressed as the rate of [^14^C]-acetate incorporation into cholesterol for μg of protein^41^.

### Preparation of cholesteryl [^14^C]-oleate HDL

Commercially available HDL (d = 1.063–1.210, Bioquote Ltd., York, UK) were incubated with cholesteryl [1-^14^C]-oleate (sp act 1.9 GBq/mmol, NEN, Boston, USA) according to a modified method of Terpstra *et al.*^42^. Briefly, radiolabeled cholesteryl oleate dissolved in toluene was evaporated under a stream of nitrogen. The tracer was resolubilised in ethanol and then added to a solution of HDL and lipoprotein-deficient serum (LPDS) at the concentration of 0.05 μCi/μg HDL. The mixture was incubated for 24 h at 37 °C in a shaking bath under nitrogen, and the [^14^C]-CE-HDL was isolated by density gradient ultracentrifugation (Beckman L8-M, Milan, Italy). The labeled HDL preparation was extensively dialysed (PBS, EDTA 1 mM, pH 7.4), protein content evaluated a sterile-filtered (0.45 mm, Millipore). Labeled HDLs, were added to a 10% LPDS DMEM medium at the concentration of 100 g/ml.

### Free cholesterol and CE content in quiescent and exponentially growing fibroblasts

Total lipids were extracted from the cells using the method of Folch *et al*^40^. Aliquots from chloroform phase were dried down under vacuum and dissolved in methanol for HPLC analysis. Separation of CEs was carried out as described^43^,^44^,^45^ using an Agilent 1100 HPLC system (Agilent, Palo Alto, CA, USA) equipped with a diode array detector and mass spectrometer in line. UV and mass spectra were recorded to confirm the identification of HPLC peaks. In some experiments cells were exposed to commercially available human HDL (density range = 1.063–1.210 g/ml, containing HDL2 and HDL3, Bioquote Ltd., York, UK), and LDL (density range = 1.019.1.063 g/ml). Prior to use, lipoprotein homogeneity was determined by agarose gel electrophoresis and the integrity of the commercially purchased HDL by evaluating the CE oxidation state as previously described^9^.

### DiI-LDL binding and DiI-HDL uptake

To evaluate the uptake of CE from HDL we utilised purified lipoproteins labeled with the fluorescent probe 1,1′-dioctadecyl-3,3,3′,3′-tetramethyl-indocarbocyanine perchlorate (DiI). To compare with the other major pathway to obtain cholesterol and then CE, the binding of LDL was evaluated. DiI is a fluorescent dye that, when excited with wavelengths around 514 nm, shows an emission peak in the range of 550 nm, suitable for viewing with a common rhodamine filter^8^,^22^. In the present study, a modified protocol was applied. In particular, cells were seeded in 35 mm glass-bottomed dishes (Ibidi, Martinsried, Germany) at a concentration of 2.0 × 10^4^cells/cm^2^ and incubated at 37 °C and 5% CO_2_ for 24, 48 and 96 h. Subsequently, cells were washed twice with PBS and once with medium 199 supplemented with 10 mM HEPES, pH 7.4, and incubated at 4 °C with DiI-LDL or DiI-HDL (Bioquote Ltd., York, UK), diluted to a concentration of 10 μg/ml for 2 h and 1 h, respectively. All lipoprotein incubations were carried out at 4 °C; at this temperature LDL bind to their receptor but internationalisation is prevented, giving the characteristic crown shape; HDL bind to the plasma-membrane protein SR-BI but only the CE component is selectively internalised. Previous experiments had shown that CE uptake is already present also at lower temperature (4 °C) and consistently increased at higher temperature (18 and 37 °C)^9^. Following incubation cells were fixed with 4% PFA for 10 min at RT and observed. For time-lapse experiments, living cells were incubated for 1 h at 4 °C with DiI-HDL, then the cells were washed with PBS and the DiI fate has been followed for up to 1 h at RT (see movies).

### LDs and plasma membrane cholesterol imaging and measurements

In order to visualise the reserve compartment of neutral lipids *in situ* (LDs), cells were cultured in 35 mm glass-bottomed dishes (Ibidi, Martinsried, Germany). After fixation with 4% PFA for at least 30 min, cells were washed in PBS and stained firstly with filipin (50 μg/ml; Sigma-Aldrich, Milan, Italy) and finally with 300 nM Nile Red (9-diethylamino-5H-benzo[*α*]phenoxazine-5-one; Fluka, Buchs, SG, Switzerland) in PBS. Filipin is a fluorescent dye that specifically stains plasma membrane cholesterol, while Nile Red (NR) stains differentially polar lipids (i.e., phospholipids) and neutral lipids (i.e., CEs and TGs). Polar lipids display a red emission, while neutral lipids a green emission. Red emission was observed with 540 ± 12.5 nm excitation and 590 LP nm emission filters. Green emission was observed with 460 ± 25 nm excitation and 535 ± 20 nm emission filters. Filipin was observed with 360 ± 20 excitation and 460 ± 25 emission filters. Filipin and Nile Red emissions were completely separated by the selected filters, so that both probes could be used in combination on the same cells^13^.

### SR-BI and BrdU immunodetection *in situ*

For SR-BI immunodetection, cells were fixed in 4% PFA for 10 min, permeabilized with 0.1% Triton X-100 in PBS supplemented with 10% FBS and incubated overnight at 4 °C with the primary anti-SR-BI polyclonal antibody (Abcam, Cambridge, UK) followed by a suitable ATTO-conjugated secondary antibody (Sigma-Aldrich, Milan, Italy) for 1 h at RT. Cells were washed with PBS and observed. In order to evaluate the proliferation state, living cells were treated with 10 μM 5-bromo-2-deoxyuridine (BrdU; Sigma-Aldrich, Milan, Italy) for 6 h. BrdU is a uridine derivative and a structural analog of thymidine, and it can be incorporated into DNA during the synthesis (S) phase of the cell cycle as a substitute for thymidine, allowing visualisation by immunostaining methods. After treatment, cells were fixed in 4% PFA for 30 min, permeabilized with 1% Triton X-100 in PBS for 15 min and, in order to break open the DNA structure, incubated first with 1 N HCL at 4 °C for 10 min and finally with 2 N HCL at RT for 30 min. Immediately after the acid washes, borate buffer (0.1 M) was added to the cells for 15 min at RT, followed by rinsing and washing, in PBS with 1% Triton X-100 at RT for 15 min. Nonspecific epitopes were blocked by incubating for 2 h in 5% goat serum in PBS with 1% Triton X-100 and then cells were incubated overnight with anti-BrdU monoclonal primary antibody (DakoCytomation, Milan, Italy) followed by a suitable ATTO-conjugated secondary antibody (Sigma-Aldrich, Milan, Italy) at RT for 1 h. Before observation, we proceeded to counterstain nuclei with 650 nM Hoechst 33258 (SigmaAldrich, Milan, Italy).

### Microscopy and imaging

Microscope observations were performed with an Olympus IX71 inverted microscope (Olympus, Tokyo, Japan) fitted with a 20x/0.7 planapochromatic objective. Twelve bit-images were captured using a cooled CCD camera (PCO Sensicam, Kelheim, Germany), electronically coupled to a mechanical shutter interposed between the 100 W Hg lamp and the microscope to limit photo bleaching. Excitation light was attenuated with a 6 or 1.5% neutral density filter. The nominal resolution of images was 0.3 microns/pixel. Quantitative analysis of images was performed with the Image Pro Plus package (Media Cybernetics, Silver Springs, MD, USA). At least 400 cells were measured individually for each experimental group.

### DNA cell cycle assay by FACS analysis

For DNA cell-cycle analysis 1.0 × 10^6^ trypsinized cells were centrifuged at 300 g for 10 min and fixed in ethanol and stored at 2–8 °C until DNA staining. Before analysis, cells were washed twice in PBS and stained with 50 μg/ml propidium iodide (Sigma-Aldrich, Milan, Italy) and 1 mg/ml RNAse (Roche, Monza, Italy) in PBS for 1 h at 2–8 °C. DNA content was measured using FACScan flow cytometer (Becton-Dickinson, Mountain View, CA, USA). The propidium iodide stained nuclei, excited at 488 nm with 200 mW laser power, was measured through a 620 ± 40 nm band-pass filter^46^. List mode files were analysed using CellQuest (Becton-Dickinson, Mountain View, CA, USA). Histograms of cell number vs linear integrated red fluorescence (proportional to DNA content) were recorded for a minimum of 10000 nuclei (excluding internal standard) at flow rates no greater than 30 to 50 events per second. Cell-cycle analysis of the DNA histograms was performed with ModFit (Becton-Dickinson, Mountain View, CA, USA). The software package includes algorithms for estimating multi-cut debris and aggregates, which were subtracted in all samples prior to cell-cycle analysis as described elsewhere^47^.

### DiO-HDL uptake by flow cytometry

To evaluate the uptake of CE from HDL by flow cytometry we utilised purified lipoproteins labeled with the fluorescent probe DiO (3,3′-dioctadecyloxacarbocyanine). In the present study, a modified protocol was applied. In particular, cells were seeded at a concentration of 2.0 × 10^4^cells/cm^2^ and incubated at 37 °C and 5% CO_2_ for 24, 48 and 96 h. Subsequently, cells were washed twice with PBS and once with medium 199 supplemented with 10 mM HEPES, pH 7.4, and incubated at 4 °C with DiO-HDL (Kalen Biomedical, MD, USA), diluted to a concentration of 10 μg/ml for 1 h. Following incubation cells were washed with PBS, trypsinized and suddenly fixed with 4% PFA for 10 min at RT. After fixation cells were washed and harvested for measurements. DIO-HDL fluorescence was observed with a BD FACS Canto II equipped with 488 ± 10 nm excitation laser and 530 ± 30 nm emission filters. All data were processed with BD Facsdiva 6.1.3 (Becton Dickinson, Mountain View, CA, USA). At least 10000 cells were evaluated for each group and each experiment was carried out at least twice. The data are reported as the weighted mean value of fluorescence evaluated in positive cells (P2) normalized to the entire population (P1 + P2).

### Protein expression by western blot analysis

The cells were lysed at 4 °C in a PBS buffer containing 10% SDS, 50 μg TRIS, 1 μM EDTA, pH 7.5, 50 μM DTT and a protease/phosphatase inhibitor cocktail, homogenised by a UP100H Compact Ultrasonic Laboratory Device (Hielscher Ultrasonic GmbH, Teltow, Germany). Protein content of each sample was determined by BCA (Sigma-Aldrich, Milan, Italy) assay^48^ and processed as previously described^8^. In particular, protein samples (30 μg/lane) were separated by electrophoresis (acrylamide precast gel; Bio-Rad, Laboratories Inc., Milan, Italy) and transferred to nitrocellulose, 0.45 μm pore size (Millipore, Milan, Italy) by standard electro-blotting procedure. The blots were pre-treated with a solution containing 5% non fat dried milk at 4 °C in TBST (50 μM TRIS-HCl, pH 7.6, 0.15 M NaCl and 0.05% Tween-20) for at least 1 h before the addition of the primary antibodies (dilution range varying from 1:200 to 1:5000) for SR-BI (anti-rabbit polyclonal antibody), pAKT (anti rabbit polyclonal antibody), AKT (anti rabbit polyclonal antibody), pERK (anti goat polyclonal antibody), ERK (anti mouse monoclonal antibody) and β-actin (anti-goat polyclonal antibody). After an overnight incubation at 4 °C, the primary antibodies were removed and appropriate horseradish peroxidase-conjugated secondary antibodies were added in a dilution range of 1:2500 to 1:10000 for at least 1 h at RT. All the antibodies were purchased by ABCAM (Cambridge, UK). Proteins were detected by enhanced chemiluminescence (Millipore, Milan, Italy) and by exposure to X-ray film (Sigma-Aldrich, Milan, Italy) for various times. Quantification of the protein bands was then accomplished by densitometric analyser (Image J software, NIH Bethesda, MA, USA).

### Statistical analysis

Data from all experiments were analysed with GraphPad Prism software version 5.00 for Windows (GraphPad, San Diego, CA, USA) and Statistica (StatSoft, Tulsa, OK, USA). All data were analysed by unpaired two-tailed t-student test or by one-/two-way ANOVA and post-hoc tests (Fischer’s LSD test) when required. Significance was set at p < 0.05.

## Additional Information

**How to cite this article**: Angius, F. *et al.* High-density lipoprotein contribute to G0-G1/S transition in Swiss NIH/3T3 fibroblasts. *Sci. Rep.* **5**, 17812; doi: 10.1038/srep17812 (2015).

## Supplementary Material

Supplementary Video 1

Supplementary Video 2

Supplementary Information

## Figures and Tables

**Table 1:** Free cholesterol and cholesteryl ester content in 3T3 fibroblasts.

Treatments	FC nmol/10^7^cell	CE nmol/10^7^cell	TC nmol/10^7^cell	CE/CT
CONFLUENT	60.90	192.63	253.46	0.76
DILUTED	60.94	501.96*	564.00*	0.89*

Free cholesterol (FC), cholesteryl ester (CE) and total cholesterol (TC), were evaluated in confluent (quiescent) and diluted (24 h proliferating) 3T3 Swiss mouse fibroblast cells.

^*^p < 0.05 vs confluent cells (t-student test).
