# Supplementary material for: High-density lipoprotein contribute to G0-G1/S transition in Swiss NIH/3T3 fibroblasts
Source: Sci Rep. 2015 Dec 7;5:17812. doi: 10.1038/srep17812 (PMC4671069; doi:10.1038/srep17812)
Supplement: Supplementary Information [file srep17812-s3.pdf]

Supplementary information

High-density lipoprotein contribute to G0-G1/S transition in Swiss NIH/3T3 fibroblasts

Fabrizio Angius, Stefano Spolitu, Sabrina Uda, Stefania Deligia, Alessandra Frau, Sebastiano Banni, Maria Collu, Simonetta Accossu, Clelia Madeddu, Roberto Serpe, Barbara Batetta

Movie legends

Movie 1. DiI-HDL in quiescent NIH/3T3 fibroblasts.

Living contact-inhibited cells at the concentration of about $4.0x10^4$cells/cm^2 (stationary growth), were incubated for 1h at 4°C with DiI-HDL, then the cells were washed with PBS and the DiI fate has been followed for up to 1h at RT.

Movie 2. DiI-HDL in proliferating NIH/3T3 fibroblasts.

Cells were diluted at $1.5x10^4$cells/cm^2. After 24h culture (exponential growth) cells were incubated for 1h at 4°C with DiI-HDL, then the cells were washed with PBS and the DiI fate has been followed for up to 1h at RT.
